# Unlocking the Entomological Collection of the Natural History Museum of Maputo, Mozambique

**DOI:** 10.3897/BDJ.9.e64461

**Published:** 2021-04-21

**Authors:** Domingos Sandramo, Enrico Nicosia, Silvio Cianciullo, Bernardo Muatinte, Almeida Guissamulo

**Affiliations:** 1 Department of Biological Sciences, Universidade Eduardo Mondlane, Maputo, Mozambique Department of Biological Sciences, Universidade Eduardo Mondlane Maputo Mozambique; 2 Department of Environmental Biology, Sapienza - University of Rome, Rome, Italy Department of Environmental Biology, Sapienza - University of Rome Rome Italy; 3 Natural History Museum of Maputo, Maputo, Mozambique Natural History Museum of Maputo Maputo Mozambique

**Keywords:** Biodiversity, dataset, digitisation, entomology, insects, specimens

## Abstract

**Background:**

The collections of the Natural History Museum of Maputo have a crucial role in the safeguarding of Mozambique's biodiversity, representing an important repository of data and materials regarding the natural heritage of the country. In this paper, a dataset is described, based on the Museum’s Entomological Collection recording 409 species belonging to seven orders and 48 families. Each specimen’s available data, such as geographical coordinates and taxonomic information, have been digitised to build the dataset. The specimens included in the dataset were obtained between 1914–2018 by collectors and researchers from the Natural History Museum of Maputo (once known as “Museu Alváro de Castro”) in all the country’s provinces, with the exception of Cabo Delgado Province.

**New information:**

This paper adds data to the Biodiversity Network of Mozambique and the Global Biodiversity Information Facility, within the objectives of the SECOSUD II Project and the Biodiversity Information for Development Programme. The aforementioned insect dataset is available on the GBIF Engine data portal (https://doi.org/10.15468/j8ikhb). Data were also shared on the Mozambican national portal of biodiversity data BioNoMo (https://bionomo.openscidata.org), developed by SECOSUD II Project.

## Introduction

More than 3000 insect species are estimated to be present in Mozambique (Ministèrio da Terra, Ambiente e Desenvolvimento Rural 2015). However, the country’s entomological diversity is poorly documented. Despite the increasing research on insect diversity, there is poor dissemination of generated data and minimal usage of these scientific findings (Ministry for Coordination of Environmental Affairs 2014). Taking into account these shortcomings, the need to document and record the country’s entomological diversity emerges.

In such a framework, the Entomological Collection of the Natural History Museum of Maputo (NHMM) could play a crucial role in documenting and disseminating data about Mozambican entomological diversity. The Natural History Museum Collections (NHMCs) are important repositories of biodiversity data and represent a fundamental system for providing references to describe the natural world (Alves et al. 2014). Conventionally, in museum collections, organisms are identified, catalogued and stored in a systematic order, representing an important and durable source of ancillary data (Sampaio et al. 2019). Moreover, they provide the source material that can be used for several biological studies (Monteiro et al. 2017) and their relevance is rapidly growing in understanding, using and protecting natural resources. Specimens in museum collections and the information available in related databases are used internationally for examining biodiversity changes related to habitat loss, climate change, biological invasions and for determining the threat status of species (Hamer 2012).

Although the NHMCs preserve pivotal information on biodiversity, they are often difficult to access. The digitisation of museum collections can contribute to overcoming this limitation by allowing easier access to museum heritage and making data on global biodiversity available to researchers and policy-makers (Drew et al. 2017). With this intent, an efficient way to tackle the digitisation of the museums records is to divide collection data by distinct subsets, representing specific taxonomic compartments (Roy and Gagnon 2016).

The NHMM houses a large zoological collection, which includes mammals, birds, reptiles, fishes, insects and other invertebrates (Natural History Museum of Maputo 2016a). The museum is part of the Eduardo Mondlane University of Maputo (UEM), both as a centre of scientific communication and as a hub for the development of biological research activities. Its mission is to preserve and promote Mozambique’s wildlife heritage, encourage scientific research on its fauna and ecosystems and, lastly, promote formal and informal environmental education, contributing to the sustainable use and management of the Country’s natural resources and ecosystems (Universidade Eduardo Mondlane 2015). Considering the poor documentation about Mozambican entomological diversity, the Entomological Collection of the NHMM created a dataset (hereafter called "the dataset") to encompass biodiversity data and to make this data available for the scientific community. The dataset records the primary biodiversity data, such as taxonomic classification, geographical coordinates of sampling site and date of collection of the specimens. A taxonomic review of the specimens was carried out and the collection sites for each occurrence were georeferenced using Google Earth 7.3 Software. The specimens of the entomological collection, included in the dataset, have been sampled in all the country’s provinces (excluding Cabo Delgado) between 1914 and 2018 by collectors and researchers from NHMM. The main contributions were made by Maria Corinta Ferreira and Gunderico da Veiga Ferreira, during their work as entomologists at the NHMM. With 176527 specimens, belonging to almost all insect orders found in the country, the Museum’s Entomological Collection is the largest specimen collection in Mozambique. It holds a pivotal value both at national and Afro-tropical Regional level (Natural History Museum of Maputo 2016b).

Thus, by making the knowledge of Mozambique's entomological diversity accessible, the dataset produced by NHMM can support researchers and policy-makers in planning strategies to manage and conserve the entomological biodiversity and its related fauna and flora.

The dataset has been developed in the framework of the *SECOSUD II Project* within the Biodiversity Network of Mozambique BioNoMo (https://bionomo.openscidata.org/bionomo) initiative, which aims to provide a tool for national aggregation of biodiversity data (SECOSUD II Italian Cooperation Project 2017b). The dataset has also benefited from the contribution of the Biodiversity Information for Development (BID) Program for sharing primary biodiversity data on the Global Biodiversity Information Facility (https://www.gbif.org/) portal through the project *Mobilizing primary biodiversity data for Mozambican species of conservation concern* (Global Biodiversity Information Facility Secretariat 2017).

## General description

### Purpose

Mobilisation of primary biodiversity data for Mozambique's entomofauna

### Additional information

The dataset is a subset of the Entomological Collection of the NHMM. The species included in the dataset were taxonomically reviewed. All dataset specimens were collected in Mozambique, during sampling expeditions conducted between 1914 and 2018, from 225 different localities. Approximately 93% of the specimens are georeferenced. The dataset includes taxonomic classification, locality name, sampling coordinates, catalogue number and collection date.

## Project description

### Title

S*ECOSUD II - Conservation and equitable use of biological diversity in the SADC region: Biodiversity Network of Mozambique initiative* and *Mobilizing primary biodiversity data for Mozambican species of conservation concern*.

### Study area description

The study area of the *SECOSUD II Project* encompasses the following countries belonging to the Southern African Development Community: Mozambique, Eswatini, South Africa and Zimbabwe. The project *Mobilizing primary biodiversity data for Mozambican species of conservation concern* was designed for Mozambique.

### Design description

The dataset was digitised in the framework of the BioNoMo initiative, as part of *SECOSUD II Project* and is one of the occurrence datasets published on GBIF through the project Mobilizing primary biodiversity data for Mozambican species of conservation concern, within the Biodiversity Information for Development Programme.

The *SECOSUD II Project* aims to consolidate the capacities of decision-makers responsible for land planning and management of natural resources. This project also aims promote and support the harmonisation of land management processes at the national, regional and international level. The main objective of the *SECOSUD II Project* is to promote biodiversity conservation and sustainable economic development in the SADC (Southern African Development Community) region, consistent with the Convention on Biological Diversity (CBD) goals (SECOSUD II Italian Cooperation Project 2017a). Project activities include support for the development of a national platform for the collection, organisation and sharing of information on biological diversity. BioNoMo activities include initialising the database of primary biodiversity data in each partner institution to create a biodiversity information network, freely available on a deputed web portal. Such web portal provides: (i) the documentation on biodiversity at national level; (ii) data on primary biodiversity; and (iii) species data such as taxonomic lists and image archives. BioNoMo aims to be a tool for national aggregation of biological diversity data, making such data available to support the development of more effective strategies for biodiversity conservation. Therefore, it is a source of information which can support scientific research and national institutions in the reporting commitments related to international convention on biodiversity conservation, such as the Convention on Biological Diversity (SECOSUD II Italian Cooperation Project 2017b). *SECOSUD II* works with research institutes involved in the management and conservation of biodiversity in the SADC region. The project works with some of the main collectors and suppliers of primary biodiversity data in the region, such as NHMM, UEM – Department of Biological Sciences, UEM – Centre of Biotechnology, Mozambican Institute for Agricultural Research, Mozambican Institute for Fishery Research, the Mozambican National Administration of Conservation Areas, Gorongosa National Park – E. O. Wilson Biodiversity Lab and Wildlife Conservation Society – Combo Project, South African National Biodiversity Institute, the University of Eswatini and the Eswatini National Trust Commission (SECOSUD II Italian Cooperation Project 2017b).

The project *Mobilizing primary biodiversity data for Mozambican species of conservation concern* aims to mobilise data on endemic and near-endemic species of plants, birds, reptiles, amphibians and fish (Biodiversity Information for Development 2019). The main objective is to increase the availability and use of biodiversity information, to support land-use decision-making (Global Biodiversity Information Facility Secretariat 2017) and biodiversity conservation strategy planning. Currently, more than 130000 occurrence records have been digitised (Biodiversity Information for Development 2019). The lead project institution in the country was the Instituto de Investigação Agrária de Moçambique (IAMM), including several additional partners involved at national and international level. Moreover, in order to promote the exchange of taxonomic knowledge, a partnership with the South African National Biodiversity Institute (SANBI) has been developed, leading to the creation of a network of experts between both countries and to the consequent capacity building in Mozambique. Another partner, the Royal Botanic Gardens, Kew, provided additional training in IUCN Red Listing through its Tropical Important Plants Area initiative. Finally, through its BioNoMo initiative, *SECOSUD II Project* provided technical assistance to the data digitising activities and has made the digitised data available via the BioNoMo portal. In addition, other partners who allowed data publishing are: Entomoteca – Ministério de Agricultura e Segurança Alimentar (MASA), National History Museum of Maputo, Universidade Eduardo Mondlane, Institute for Fishery Research and South African Aquatic Biodiversity Institute (SAIAB) (Biodiversity Information for Development 2019).

### Funding

The Italian Agency for Development Cooperation funds *SECOSUD II Project*. European Union and Global Biodiversity Information Facility funded *Mobilizing primary biodiversity data for Mozambican species of conservation concern*.

## Sampling methods

### Study extent

**Figure 1.**
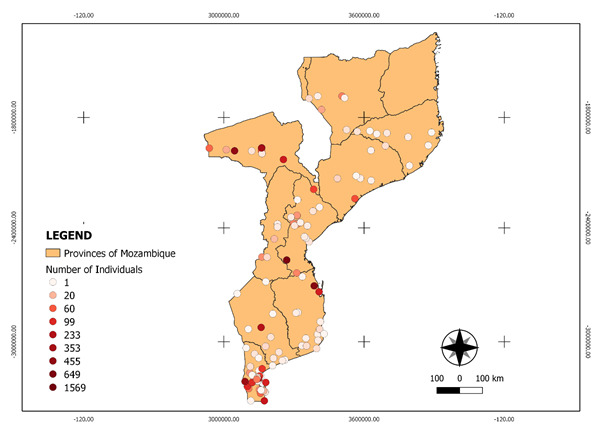
Distribution map of specimens' occurrence sites.

Sampling occurred in nine (9) provinces of the Country (Maputo, Gaza, Inhambane, Manica, Sofala, Tete, Zambezia, Nampula and Niassa). No records were collected in Cabo Delgado Province (Fig. 1).

### Sampling description

Samples from the Entomological Collection of NHMM were collected between 1914 and 2018 in 225 localities. The main contributor to the collection was Maria Corinta Ferreira Fontes de Melo Ferreira (1922–2003), during her work as resident entomologist at the NHMM and Gunderico da Veiga Ferreira, an entomologist for the Board of Geographical Missions and Colonial Research. In 1949, Maria Corinta Ferreira established a programme for collecting insects in the wood sawmills and forests of the Maputo Region. Consequently, the programme was extended (mainly in 1965 and 1973) to other southern provinces, with the intent of enriching and diversifying the museum’s entomological collection (Antunes 2016).

In the most recent years, the collection was expanded through contributions from National Parks and Reserves; the majority of specimens was donated by Gorongosa National Park and the Maputo Special Reserve. Field expeditions, conducted by the NHMM, have also led to an increase in the number of specimens, particularly in the Coleoptera and Lepidoptera orders.

### Quality control

The data quality control was supported by national and international entomology experts, which validated the taxonomic classification of specimens.

### Step description

In the framework of the BioNoMo initiative and the *Mobilizing primary biodiversity data for Mozambican species of conservation concern* project, assistants and staff of data provider institutions were trained on biodiversity database creation, digitisation and management.

The dataset was developed through the digitisation of labels and field cards of the specimens in the Entomological Collection of NHMM. The data included in the dataset were cleaned with an exhaustive review of existing museum material. Such review was supported by entomology specialists from the Sapienza – University of Rome, by performing a taxonomic verification of each specimen collected. Specimens without a reliable taxonomic classification were not included in the dataset. Thus, a revision of the cataloguing records of the included specimens (such as catalogue number, collector, data, location of collection, storage and the status of maintenance of the specimens) was performed. In order to automatically update the dataset through an international biodiversity database, such as the Catalogue of Life (https://www.catalogueoflife.org/) and the Encyclopedia of Life (https://eol.org/), taxonomic classification was updated and validated using R Statistical Program version 3.4.2., which allowed us to compare our data with other entomological databases. Data were digitised through SPECIFY Version 7.

Approximately 93% of specimens were georeferenced according to the guidelines of Chapman and Wieczorek (2020). Using the Georeferencing Calculator tool, the point-radius method was adopted as a practical solution for the georeferencing of descriptive localities. This method has been chosen to facilitate the management of uncertainties related to the georeferencing of the older samples (Wieczorek et al. 2004).

In addition, maps and gazetteers were used to further refine the georeferencing of the sampling locations by providing coordinates and spatial boundaries for the sites described in the field card of each specimen. The geographic coordinates were determined using Google Maps. Decimals of geographic coordinates were based on the World Geodetic System 84 (WGS84) datum.

The georeferencing process applied is consistent with the requirements of the Darwin Core standard on which the dataset has been built. The Darwin Core standard is an open access ensemble of rules and definitions to facilitate the digital sharing of information about biological diversity. Darwin Core is based mainly on the concept of taxa, their occurrence in nature, as documented by observations, specimens, samples and related information (Wieczorek et al. 2010).

## Geographic coverage

### Description

**Figure 2.**
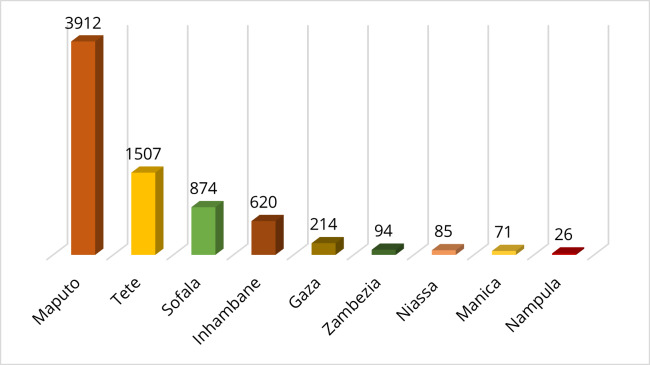
Number of insect specimens from each of Mozambique’s Provinces involved.

The dataset covers all Mozambique’s provinces, except Cabo Delgado. The specimens were collected in different provinces of Mozambique as follows: Maputo (3912), Tete (1507), Sofala (874), Inhambane (620), Gaza (214), Zambezia (94), Niassa (85), Manica (71) and Nampula (26) (Fig. 2). Georeferencing could not be carried out for 564 specimens.

## Taxonomic coverage

### Description

**Figure 3.**
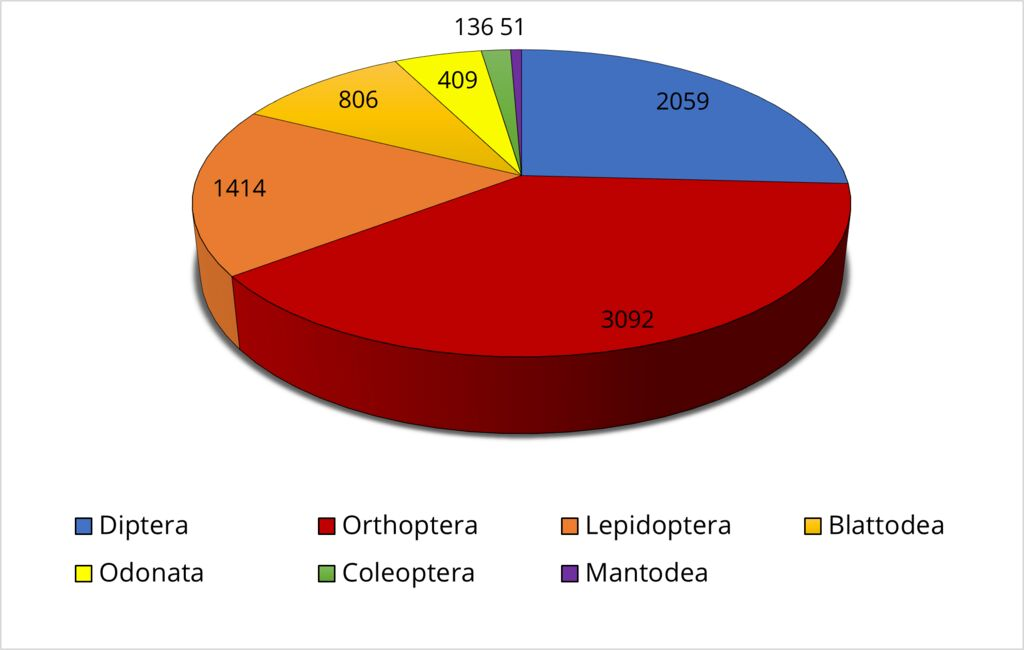
Number of insect specimens per order presented in the dataset

The dataset includes 7967 specimens from seven orders, 48 families and 409 species. Orthoptera is the most represented order (39% of the specimens), followed by Diptera (26%) and Lepidoptera (18%). The remaining part of the specimens belongs to the orders Blattodea, Odonata, Coleoptera and Mantodea, which account for 10%, 5%, 2% and 0.60% of the data, respectively (Fig. 3).

### Taxa included

**Table:** 

Rank	Scientific Name	
kingdom	Animalia	
phylum	Arthropoda	
class	Insecta	
order	Diptera	
family	Bombyliidae	
family	Calliphoridae	
family	Conopidae	
family	Glossinidae	
family	Hippoboscidae	
family	Muscidae	
family	Sarcophagidae	
family	Syrphidae	
family	Tabanidae	
order	Orthoptera	
family	Acrididae	
family	Gryllidae	
family	Gryllotalpidae	
family	Pamphagidae	
family	Pyrgomorphidae	
family	Tetrigidae	
family	Tettigoniidae	
order	Lepidoptera	
family	Arctiidae	
family	Crambidae	
family	Erebidae	
family	Eupterotidae	
family	Geometridae	
family	Hesperiidae	
family	Lasiocampidae	
family	Limacodidae	
family	Nymphalidae	
family	Lymantriidae	
family	Noctuidae	
family	Notodontidae	
family	Papilionidae	
family	Pieridae	
family	Saturniidae	
family	Sphingidae	
order	Blattodea	
family	Blaberidae	
family	Blattidae	
family	Corydiidae	
family	Ectobiidae	
order	Odonata	
family	Aeshnidae	
family	Chlorocyphidae	
family	Coenagrionidae	
family	Gomphidae	
family	Lestidae	
family	Libellulidae	
order	Coleoptera	
family	Carabidae	
family	Cerambycidae	
order	Mantodea	
family	Empusidae	
family	Mantidae	
family	Tarachodidae	

## Temporal coverage

### Notes

The temporal range of the records is between 1914 and 2018. The main years of data collection were 1949, 1965 and 1973, during which 1219, 878 and 811 specimens were recorded, respectively.

## Usage licence

### Usage licence

Creative Commons Public Domain Waiver (CC-Zero)

## Data resources

### Data package title

Colecção Entomológica do Museu de História Natural de Maputo

### Resource link

The dataset is freely accessible on the GBIF Engine data portal (https://doi.org/10.15468/j8ikhb). Data were also shared on the national portal BioNoMo (https://bionomo.openscidata.org/bionomo/advsearch) developed by the SECOSUD II Project.

### Number of data sets

1

### Data set 1.

#### Data set name

Colecção Entomológica do Museu de História Natural de Maputo

#### Data format

Darwin Core Archive format

#### Number of columns

48

#### Character set

UTF-8

#### Download URL

www.gbif.org/dataset/c61f14e7-95fd-4785-8d08-3ea6507c162e

#### Data format version

1.0

#### Description

The dataset **Colecção Entomológica do Museu de História Natural de Maputo** (Sandramo and Chuquela 2020) is composed of 7967 specimens, belonging to seven orders, 48 families and 409 species. A total of 53 species listed in the dataset are classified in the IUCN Red List of Threatened Species (International Union for Conservation of Nature 2020). Fifty species are in the “Least Concern” category, while the species *Acisoma panorpoides* (Clausnitzer et al. 2018), *Amblyphymus adspersus* (Hochkirch et al. 2018) and *Empusa fasciata* (Shcherbakov and Battiston 2020) are classified as “Endangered”, “Near Threatened” and “Data Deficient”, respectively.

**Data set 1.** 

Column label	Column description
ID	Identifier for the collection from which the record was derived
Type	The nature of the resource
Language	The language of the resource
Institution Code	The acronym used by the institution (Museu De História Natural de Maputo) having custody the resource
Collection Code	The acronym identifying the collection or dataset from which the record was derived
Dataset Name	The name identifying the dataset
Owner Institution Code	Acronym used by the institution having ownership of the resource
Basis of Record	The specific nature of the data recorded
Occurrence ID	The identifier code of the occurrence
Catalogue Number	The identifier number for the record within the collection
Recorded by	Collectors or obervers responsible for recording the original occurrence
Individual count	The number of individuals occurring
Sex	The sex of individual occurring
Life stage	The age class of the individual occurring
Occurrence status	Presence or absence of the taxon in Mozambique
Preparations	Preparation and preservation method of the specimen
Sampling protocol	The description of the method used during the occurrence
Event date	The date of the occurrence
Year	The year of the occurrence
Month	The integer month of the occurrence
Day	The integer month's day of the occurrence
Habitat	Habitat's description of the occurrence
Continent	The full name of the occurrence's continent
Country	The full name of the occurrence's country
Country code	The standard code of the occurrence's country
State or Province	The country's administrative region (province) of the occurrence
Locality	The specific location of the occurrence
Maximum elevation metres	The altitude above sea level (in metres) of the occurrence
Georeference source	The sources used to georeference the location
Decimal latitude	The geographic latitude, expressed in decimal degrees, of the geographic centre of the location
Longitude	The geographic longitude, expressed in decimal degrees, of the geographic centre of the location
Geodetic datum	The spacial reference system underlying the geographic coordinates
Coordinate uncertainty metres	The horizontal distance (in metres) from geographic coordinates describing the smallest circle containing occurrence's location
Identified by	The person who assigned the Taxon to the subject
Scientific name	The scientific name (genus, specific epithet, authorship and date) of the specimen
Taxonomic status	The status of the use of the reported scientific name
Kingdom	The scientific name of the kingdom to which the specimen belongs
Phylum	The scientific name of the phylum to which the specimen belongs
Class	The scientific name of the class to which the specimen belongs
Order	The scientific name of the order to which the specimen belongs
Family	The scientific name of the family to which the specimen belongs
Genus	The scientific name of the genus to which the specimen belongs
Subgenus	The scientific name of the subgenus to which the specimen belongs
Specific epithet	The species epithet of the specimen's scientific name
Infraspecific epithet	The terminal infraspecific epithet of the specimen's scientific name
Taxon rank	The taxonomic rank of the specimen
Scientific name authorship	The authorship information for the scientific name, according to the nomenclature code
Vernacular name	The common vernacular name
